# Comprehensive Analysis of Preeclampsia-Associated DNA Methylation in the Placenta

**DOI:** 10.1371/journal.pone.0107318

**Published:** 2014-09-23

**Authors:** Tianjiao Chu, Kimberly Bunce, Patricia Shaw, Varsha Shridhar, Andrew Althouse, Carl Hubel, David Peters

**Affiliations:** 1 Department of Obstetrics, Gynecology and Reproductive Sciences, University of Pittsburgh, Pittsburgh, Pennsylvania, United States of America; 2 Magee-Womens Research Institute, University of Pittsburgh, Pittsburgh, Pennsylvania, United States of America; VU University Medical Center, Netherlands

## Abstract

**Background:**

A small number of recent reports have suggested that altered placental DNA methylation may be associated with early onset preeclampsia. It is important that further studies be undertaken to confirm and develop these findings. We therefore undertook a systematic analysis of DNA methylation patterns in placental tissue from 24 women with preeclampsia and 24 with uncomplicated pregnancy outcome.

**Methods:**

We analyzed the DNA methylation status of approximately 27,000 CpG sites in placental tissues in a massively parallel fashion using an oligonucleotide microarray. Follow up analysis of DNA methylation at specific CpG loci was performed using the Epityper MassArray approach and high-throughput bisulfite sequencing.

**Results:**

Preeclampsia-specific DNA methylation changes were identified in placental tissue samples irrespective of gestational age of delivery. In addition, we identified a group of CpG sites within specific gene sequences that were only altered in early onset-preeclampsia (EOPET) although these DNA methylation changes did not correlate with altered mRNA transcription. We found evidence that fetal gender influences DNA methylation at autosomal loci but could find no clear association between DNA methylation and gestational age.

**Conclusion:**

Preeclampsia is associated with altered placental DNA methylation. Fetal gender should be carefully considered during the design of future studies in which placental DNA is analyzed at the level of DNA methylation. Further large-scale analyses of preeclampsia-associated DNA methylation are necessary.

## Introduction

Preeclampsia is a hypertensive disorder that affects 5–7% of all pregnancies and is one of the major obstetrical complications resulting in fetal morbidity and mortality [1]. It is defined by maternal arterial pressure >140/90 mmHg and proteinuria >300 mg/dL/24 h after 20 weeks gestation. Although preeclampsia is a complex phenotype with underlying pathobiology that likely involves multiple systems in both mother and fetus, it is thought to be partly caused by disruption or alteration of the highly regulated and ordered processes underlying trophoblast invasion and placentation. The result of this is that placentas from preeclamptic pregnancies are poorly perfused due to inadequate remodeling of the spiral arteries [2], [3], [4].

Numerous studies have identified altered gene expression in the placentas of mothers affected by preeclampsia [5], [6], [7], [8], [9]. Preeclampsia-specific altered gene expression has also been reported in chorionic villus samples obtained in early gestation from pre-symptomatic mothers who went on to develop preeclampsia [10], [11]. Furthermore, it has also been shown that alterations in expression patterns are not confined to the placenta and can be observed in circulating maternal white blood cell populations [12].

Although the relationship between DNA methylation and gene expression at the level of transcription is complex, there is strong evidence that altered DNA methylation, particularly within the regulatory regions of expressed genes, has a significant influence on transcription [13], [14], [15]. Given the strong evidence that gene expression is altered in preeclamptic pregnancies, it is reasonable to hypothesize that preeclampsia may also be associated with altered DNA methylation in key regulatory regions. Indeed, in 2010, it was reported that early onset (<34 weeks gestation), but not late onset (>34 weeks gestation), preeclampsia is associated with altered DNA methylation [16]. This study was somewhat limited by the number of individuals (n = 4 per group) that were analyzed. In a more recent larger study the same group found that DNA methylation is altered in placental samples from individuals suffering with early onset preeclampsia (EOPET) when compared to gestational age-matched karyotypically normal samples obtained from women who suffered pregnancy loss or early labor for a variety of reasons [17].

Given our relatively limited understanding of epigenetic alterations in preeclampsia, it is important that further studies are undertaken so that reported associations between DNA methylation and preeclampsia may be verified and expanded upon. Therefore we have undertaken an analysis of DNA methylation in the context of preeclampsia using a commercially available microarray that is able to simultaneously assess DNA methylation levels at 27,000 CpG dinucleotides.

## Materials and Methods

### Samples

All patients provided written informed consent for use of their samples and de-identified clinical data under the umbrella of the Preeclampsia Program Project (PEPP study) and related ancillary studies. These women delivered at Magee-Womens Hospital within the years 1997–2007, and were recruited from clinics and private practices. This protocol was approved by the University of Pittsburgh Institutional Review Board (Protocol # IRB 0404159). Clinical and demographic characteristics of the individuals recruited to take part in this study are shown in Table 1.

**Table 1:** Sample Demographics and Clinical Characteristics.

	Normal pregnancy (n = 24)	Preeclamptic pregnancy (n = 24)	P-value
Maternal age (years)	29.3±5.4	27.9±7.2	0.549
Gestational age at delivery (weeks)	39.3±1.2	35.0±4.0	<0.001*
Maternal race (n, %)			
White	21 (86.3%)	19 (79.2%)	0.589
African-American	3 (13.7%)	4 (16.7%)	
Other	0 (0.0%)	1 (4.2%)	
Nulliparous (n)	23	24	0.289
Pre-pregnancy BMI	24.1±4.5	25.3±4.0	0.21
Systolic BP<20 weeks (mm Hg)	114.1±8.0	118.2±10.4	0.115
Diastolic BP<20 weeks (mm Hg)	69.5±5.5	71.1±6.5	0.242
Systolic BP at delivery (mm Hg)	122.6±11.3	152.0±8.6	<0.001*
Diastolic BP at delivery (mm Hg)	73.3±9.3	96.2±7.9	<0.001*
Proteinuria (n)	0	24	<0.001*
Uric acid (mg/dL)	ND	6.2±0.9	
Cesarean delivery (n, %)	15 (62.5%)	15 (62.5%)	1.0
Birth weight (g)	32288.1±588.6	2314.8±1121.8	0.002*
Birth weight centile (%)	49.3±30.6	34.4±28.9	0.095
Female baby gender (n, %)	13 (54.2%)	10 (41.7%)	0.56

Data presented as Mean ± Standard Deviation as marked.

ND  =  not determined.

A panel of clinicians met monthly to adjudicate the pregnancy outcome diagnosis of all the women. Preeclampsia was defined as gestational hypertension with proteinuria in accordance with the National High Blood Pressure Education Program Working Group Report on High Blood Pressure in Pregnancy [18]. Gestational hypertension was defined as persistent, new onset hypertension (systolic ≥140 mm Hg and/or diastolic ≥90 mm Hg) appearing after 20 weeks' gestation. Proteinuria was defined as ≥300 mg of protein in a 24 hour urine collection, a dipstick of 2+, a catheterized sample of 1+, or protein:creatinine ≥0.3. To ensure that preeclamptic women had the specific disease of interest, our research definition also required gestational hyperuricemia (≥1 standard deviation above reference values for the gestational age (e.g. term, >5.5 mg/dL)) [19]. Hyperuricemia is thought to minimize the number of women with hypertension in pregnancy who are misclassified as preeclamptics [20]. Women with uncomplicated (“normal”) pregnancy were normotensive and without proteinuria throughout gestation, and delivered healthy babies in the absence of infection.

All participants had no prior history of renal or vascular disease, and were non-smokers (<100 cigarettes in lifetime). Status of tobacco smoking was determined by self-report; these data were obtained during the pregnancy or immediately postpartum to minimize the potential problems caused by changing smoking habits during pregnancy. In a previous study of our patient population, there was no significant discordance between self-report of smoking status and maternal plasma cotinine concentration using a cutoff value of ≥2.0 ng/ml (P = 0.49), with 82% agreement and κ coefficient of 0.64 [21]. Pre-pregnancy weight, self-reported at enrollment, and measured height were used to calculate pre-pregnancy body mass index (BMI; weight [kg]/height [m^2^]). Maternal race was by self-report at enrollment.

Placental biopsies were obtained immediately after delivery from the maternal side of the placenta, in regions free of infarcts between the placental margin and cord insertion. Decidua was removed by blunt dissection. The tissue was immediately transported in saline to the laboratory, flash frozen in liquid nitrogen and stored at −80°C for later use.

### DNA Isolation

To isolate DNA, 180-µL buffer ATL (from Qiagen's DNeasy Blood and Tissue kit, Qiagen, Valencia, CA) and a 5-mm steel bead were added to each placenta sample. The samples were then homogenized in a TissueLyser (Qiagen) for 20 s at 30 Hz. The DNA was then purified using the DNeasy Blood and Tissue kit as per the manufacturer's protocol.

### Infinium Microarray Analysis and Determination of Methylation Status

DNA samples were prepared for microarray analysis and methylation status determined as previously described (Bunce, et al, 2012). P values were adjusted using Benjamini and Hochberg's method to control the false discovery rate (FDR) at 10%. DNA methylation data has been deposited at the Gene Expression Omnibus repository (GE59274).

### Targeted Bisulfite Sequencing

DNA was bisulfite converted using Zymo's EZ DNA Methylation Gold kit as per manufacturer's protocol (Zymo Research, Irvine, CA). Bisulfite specific primers were designed using Methprimer (www.urogene.org/methprimer) or BiSearch (http://bisearch.enzim.hu). PCR products were amplified as separate reactions using Pfu Turbo Cx (Agilent Technologies, Santa Clara, CA), Platinum Taq (Life Technologies, Carlsbad, CA) or ZymoTaq (Zymo Research). Primer sequences, melting temperatures (Tm) and cycling conditions are shown in Tables S1A and S1B in File S1. Each amplicon was visualized on a 2% agarose gel. Amplicons were pooled by sample by volume and pools were purified using Agencourt AMPure XP Beads (Beckman Coulter, Brea, CA) per manufacturer's protocol. Sample pools were run on Bioanalyzer to confirm appropriate size distribution and sufficient quantity of DNA for library prep. Libraries were prepared using Illumina's TruSeq DNA Sample Prep Kit and sequenced on a HiSeq 2500 as per manufacturer's protocols (Illumina, San Diego, CA).

### Sequenom Epityper/Quantitative Methylation Analysis

Quantitative Methylation Analysis was performed as previously described [22]. Primer sequences are presented in Table S1C in File S1.

### Statistical Analysis

We compared the% methylation between cases versus normal pregnancy controls or samples from normal pregnancies involving males versus female offspring, and P-values were adjusted using the false discovery rate. Adjusted p-values less than 0.1 were considered statistically significant.

### Hierarchical Clustering and Multidimensional Scaling

We applied two different types of unsupervised learning algorithms (agglomerative hierarchical clustering and multi-dimensional scaling) on the methylation data to visualize the similarity of the methylation profiles across the samples. For hierarchical clustering, we used the Ward's minimum variance method, and chose respectively the Euclidean distance between the methylation rate of all probes, as well as 1– Pearson's correlation between the log ratio of the signal B to signal A of all probes, as the distance between two samples. The same two types of distance metric were also used in the multi-dimensional scaling analysis of the methylation data. Both types of unsupervised learning analyses were performed using the statistical computing program R.

## Results

### Patient demographics

The clinical and demographic data for the 48 subjects who provided placental samples are presented in Table 1. As expected based on definitions, women with preeclampsia had proteinuria, elevated uric acid, and higher systolic and diastolic blood pressures at delivery. Maternal age, race, parity (1 uncomplicated pregnancy was multiparous), mode of delivery and blood pressure before 20 weeks were not statistically different between the groups. Overall, the gestational age at delivery for preeclamptic women was significantly lower than the uncomplicated pregnancy group. Infant birth weights were significantly lower in the preeclamptic group. Birth weight percentiles were lower, but not significantly so, compared to controls.

### Placental Methylation Signatures Correlate Strongly With Fetal Gender

**Figure 1:**
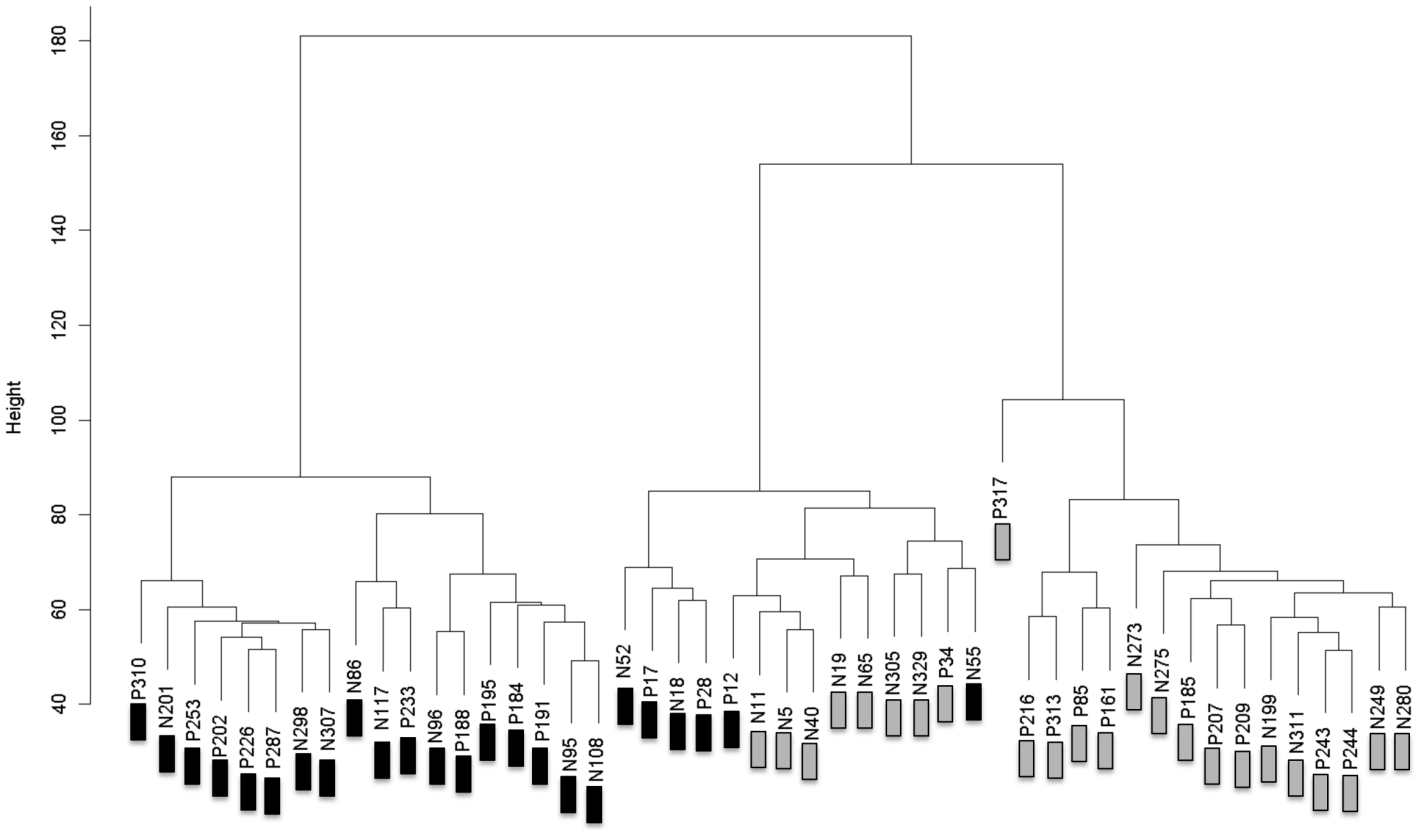
Hierarchical Clustering of all DNA Methylation Data. Samples marked with “P” and “N” represents “normal” and “preeclampsia” samples respectively. Samples marked with black and grey boxes are from pregnancies in which the fetus was male and female respectively. Data points from all chromosomes were included.

We first compared DNA methylation patterns in placental tissue samples from preeclampsia cases with normal controls by Infinium microarray. This identified a small number of differentially methylated CpG sites (Table S2A and S2B in File S1), the majority of which were hypomethylated in preeclampsia samples. We then performed an unsupervised hierarchical clustering analysis of all data with the goal of identifying patterns of methylation between sub-groups of preeclampsia and normal control samples. This revealed that DNA methylation was strongly associated with fetal gender irrespective of disease state (Figure 1). This finding strongly influenced subsequent analysis of our data in that we controlled for the influence of fetal gender either by analyzing male and female samples separately or by excluding X-chromosome specific probes as described below.

### Fetal Gender-Dependent Preeclampsia-Associated Changes in Placental DNA Methylation

Based upon the above findings we reanalyzed our data by considering pregnancies involving male and female fetuses separately. This resulted in the identification of a considerably larger number of differentially methylated loci when FDR was controlled at 10%. CpG sites with statistically significant differences of >10% methylation between preeclampsia and control samples are shown in Tables 2 and 3. (Note that statistically significant differences of <10% methylation between preeclampsia and control samples are shown in S3A and S3B).

**Table 2:** Fetal Gender-Dependent Preeclampsia-Associated Changes in Placental DNA Methylation.

Illumina Probe Identifier	% methylation PE	% methylation Norm	Combined p value	Combined Adj. p value	Chr	Symbol	Distance to TSS	CPG Island
cg14696348	13	33	2.6E-06	0.05	16	MC1R	509	TRUE
cg00779924	42	62	1.0E-03	0.09	10	FLJ45983	806	TRUE
cg10994126	49	67	4.0E-07	0.05	1	PAPPA2	191	FALSE
cg18279742	40	57	1.3E-06	0.05	16	RPS2	876	TRUE
cg24813163	16	30	9.9E-04	0.09	X	FLJ30058	85	TRUE
cg19771541	17	31	5.1E-04	0.07	X	MID2	43	TRUE
cg19410364	58	71	1.7E-04	0.05	12	P2RX4	447	TRUE
cg26304237	40	53	1.4E-03	0.10	1	DNAJC6	46	TRUE
cg09450238	27	40	1.0E-04	0.05	14	BTBD6	775	TRUE
cg04848452	15	28	7.9E-04	0.08	3	EIF1B	372	TRUE
cg05358404	35	47	3.3E-05	0.05	20	RTEL1	697	TRUE
cg17132967	29	41	5.8E-05	0.05	19	ZNF83	666	TRUE
cg15271616	57	69	2.7E-04	0.06	9	RUSC2	391	TRUE
cg07156669	55	67	1.2E-04	0.05	17	CPD	739	TRUE
cg10045881	63	74	1.2E-03	0.09	1	CHI3L2	10	FALSE
cg13283751	36	48	2.1E-04	0.05	6	GPX5	22	FALSE
cg10210238	67	78	6.6E-04	0.08	9	CDKN2B	NA	TRUE
cg09816471	53	64	7.7E-04	0.08	16	SNN	457	TRUE
cg25839227	49	61	1.3E-04	0.05	17	ABI3	913	FALSE
cg18530324	47	59	3.5E-04	0.06	18	KIAA0427	435	TRUE
cg23065097	37	48	9.0E-04	0.09	2	FKBP1B	801	TRUE
cg00503840	48	59	2.3E-04	0.05	7	DLX5	NA	TRUE
cg19884658	46	57	1.2E-03	0.09	1	KLHL21	1339	TRUE
cg11283860	52	62	4.0E-04	0.07	1	SLC45A1	1440	TRUE
cg26170660	54	64	1.3E-03	0.10	6	GPX5	188	FALSE
cg14023451	56	66	2.3E-04	0.05	6	GPLD1	37	FALSE
cg11824111	24	34	2.0E-04	0.05	X	DKC1	474	TRUE
cg17638468	40	50	4.9E-06	0.05	3	CD200R1	125	FALSE

CpG sites at which samples from normal controls (Norm) are more highly methylated (10% or greater) than those from affected individuals (PE) are shown. Significant adjusted p values (FDR controlled at 10%) were only identified in pregnancies in which the fetal gender was female.

**Table 3:** Fetal Gender-Dependent Preeclampsia-Associated Changes in Placental DNA Methylation.

Illumina Probe Identifier	% meth PE	% meth Norm	Combined pvalue	Combined Adj. pvalue	Chr	Symbol	Distance to TSS	CPG Island
cg23717696	77	57	2.9E-05	0.05	X	ITGB1BP2	432	FALSE
cg09528351	54	40	1.9E-04	0.05	17	PIK3R5	432	FALSE
cg07864297	40	26	1.3E-04	0.05	14	ESRRB	157	FALSE
cg05275605	59	47	1.1E-03	0.09	21	C21orf123	102	TRUE
cg07017706	58	46	5.8E-04	0.08	12	K6IRS3	988	FALSE
cg14358743	65	54	1.2E-03	0.09	13	GAS6	1223	TRUE
cg14886269	41	30	1.0E-03	0.09	1	TNFRSF18	162	TRUE
cg01560871	62	51	5.6E-04	0.08	10	C10orf27	267	FALSE
cg17229388	66	55	1.4E-04	0.05	13	MGC35169	128	TRUE
cg16698623	71	61	6.4E-04	0.08	10	MGMT	NA	TRUE
cg01910481	74	63	7.1E-05	0.05	20	PLUNC	266	FALSE

CpG sites at which samples from affected individuals (PE) are more highly methylated (10% or greater) than those from normal controls (Norm) are shown. Significant adjusted p values (FDR controlled at 10%) were only identified in pregnancies in which the fetal gender was female.

**Figure 2:**
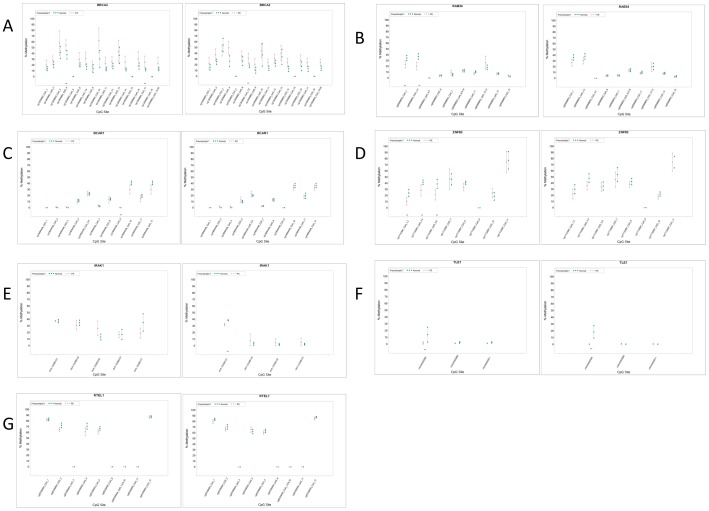
Targeted Analysis of Differentially Methylated Loci in Preeclampsia. Locus-specific analysis of CpG methylation in normal controls and preeclampsia samples was carried out by Sequenom Epityper (TLE1, IRAK1) (n = 24 controls and n = 24 PE samples) or high-throughput bisufite sequencing (n = 12 controls and n = 12 PE). Samples from pregnancies involving female (left panel) and male (right panel) fetuses were analyzed separately. Statistically significant differences in% methylation between samples are marked with **. Genomic coordinates corresponding to values on the x-axis can be found in Table S6A in File S1.

These differences were only found in samples from pregnancies in which the fetal gender was female. All significant differentially methylated sites involved changes in the frequency of methylated CpG sites of <20%. No significant differentially methylated CpG sites were found in placentas from pregnancies where the fetal gender was male. These results were confirmed for a subset of CpG sites in distinct genes by Sequenom Epityper analysis and high-throughput bisulfite sequencing (Figure 2). Notably, a number of these sites were significantly differentially methylated in samples of both fetal genders when single CpG loci were analyzed in this fashion (Figure 2).

### DNA Methylation is Altered in Early Onset Preeclampsia

Previous studies have reported that significant differences in DNA methylation exist in placental tissue from mothers with EOPET (<34 weeks gestation) when compared to normal controls who delivered at term. These previous studies, which controlled for gender bias by removing all X chromosome data points prior to analysis, reported no differences when comparing late onset preeclampsia samples (>34 weeks gestation) with normal pregnancy controls. To be consistent with these previous study designs we therefore reanalyzed our data, after excluding X chromosome-specific probe results, to compare EOPET (n = 9) samples with all normal controls (n = 24). As shown in Table 4 (and S4A in File S1), we identified 49 CpG sites whose methylation levels are significantly altered in placental tissue from EOPET cases versus normal controls when FDR is controlled at 10%. We found far more CpG sites (78%) that were hypomethylated in EOPET samples when compared to normal controls than visa versa. Of the genes identified as being differentially methylated between EOPET and controls, 28 overlapped with those listed in Tables 2 and 3. Differentially methylated CpGs, albeit far fewer, were also identified when comparing late onset samples with controls (Table 5). We also performed similar analyses in a gender-dependent fashion. Specifically, EOPET samples were compared with normal controls and pregnancies involving female and male fetuses were analyzed separately. Using this approach we found only minimal overlap (3 sites out of a possible 24) between significantly altered CpG sites in pregnancies involving male versus female fetuses. Gender-dependent differences in methylation patterns were also observed between late onset preeclampsia cases and controls but only in pregnancies involving female (and not male) fetuses (Table S4B in File S1).

**Table 4:** DNA Methylation Changes in Early Onset Preeclampsia (EOPET) Versus Normal Controls (Norm).

Illumina Probe Identifier	PE<34 weeks	Norm	Combined pvalue	Combined adjusted pvalue	Chr	Symbol
cg14696348	11	34	1.20E-11	3.32E-07	16	MC1R
cg10994126	46	65	5.59E-09	7.71E-05	1	PAPPA2
cg15271616	55	68	1.93E-08	0.000133122	9	RUSC2
cg09450238	24	39	1.38E-07	0.00054516	14	BTBD6
cg00503840	46	60	3.54E-07	0.000976753	7	DLX5
cg26304237	35	50	5.08E-07	0.00107717	1	DNAJC6
cg00410921	26	37	9.28E-07	0.001599147	15	CKMT1B
cg27360098	35	23	1.24E-06	0.001904068	7	ELN
cg02089348	48	58	2.40E-06	0.002470369	4	TMEM129
cg26954174	41	51	2.89E-06	0.002604173	16	CARD15
cg02110963	43	53	4.34E-06	0.003510057	6	MGC40222
cg03752885	32	42	4.27E-06	0.003510057	19	DAPK3
cg15503752	39	52	4.57E-06	0.003510057	17	ST6GALNAC1
cg18279742	40	54	5.57E-06	0.003941476	16	RPS2
cg18328334	29	39	6.66E-06	0.004477123	2	TNS1
cg19385139	40	51	7.15E-06	0.004587805	13	COL4A2
cg04502814	29	43	7.73E-06	0.004750353	5	SEPP1
cg00520135	62	72	1.57E-05	0.007208179	15	TPM1
cg11283860	51	62	3.46E-05	0.011779235	1	SLC45A1
cg07824742	69	57	3.77E-05	0.011815535	9	DBH
cg10045881	61	74	3.57E-05	0.011815535	1	CHI3L2
cg10590292	56	71	4.25E-05	0.01288429	12	BIN2
cg23917399	9	21	4.56E-05	0.013248486	5	TNFAIP8
cg04765277	53	68	6.26E-05	0.016138871	10	FLJ45983
cg12840719	36	47	6.33E-05	0.01615595	9	CDKN2A

% methylation levels for top 25 most highly significant differentially methylated CpG sites in EOPET (PE<34 weeks, n = 9) and normal controls (Norm, n = 24). Adjusted p values are shown with FDR controlled at 10%. Full table of all 49 significant CpG sites is shown in Table S4A (in File S1).

**Table 5:** DNA Methylation Changes in Late Onset Preeclampsia Versus Normal Controls.

Illumina Probe Identifier	PE>34 weeks	norm	Combined pvalue	Combined adj. pvalue	Chr	Symbol
cg00250430	34	45	0.0003	0.0760	9	DMRT2
cg03214697	22	42	0.0007	0.0958	22	SEC14L4
cg04576021	67	56	0.0002	0.0746	6	HLA-DOB
cg21820677	12	24	0.0003	0.0760	4	GABRA2
cg25484904	67	77	0.0008	0.0982	4	FLJ21511

% methylation levels for late onset preeclampsia (PE>34 weeks, n = 14) and normal controls (norm, n = 24) are shown. Adjusted p values are shown with FDR controlled at 10%.

### Gestational Age does not Substantially Influence DNA Methylation

Given previously published evidence that gestational age at time of delivery is a major contributor towards altered DNA methylation [16], [17], we directly compared preeclampsia placental samples obtained at delivery before and after 34 weeks gestation after removal of X chromosome-specific probe data. Only a single CpG site, MC1R, was found to be differentially methylated between <34 and>34 week samples with FDR controlled at 10%. Hierarchical clustering supported this finding and multidimensional scaling (MDS) analyses that showed no clear evidence of clustering of early versus late onset preeclampsia samples (Figure S1). A similar lack of gestational age-related clustering was also observed when normal controls were analyzed alone (not shown). Taken together these findings indicate minimal impact of gestational age at delivery, within the third trimester, on DNA methylation.

### Lack Correlation Between Gene Expression and DNA Methylation in EOPET

Given the fact that changes in DNA methylation within gene regulatory sequences are known to influence gene expression, we analyzed a publicly available gene expression microarray data set in which placental tissue samples from pregnancies affected with EOPET were compared to normal control tissues (http://www.ncbi.nlm.nih.gov/geo/query/acc.cgi?targ=self&form=html&view=quick&acc=GSE44711). We hypothesized that reduced CpG methylation in a given sample cohort (preeclampsia or control) identified in our own data would correlate with an increase in transcription. We could find no overlap between genes containing differentially methylated CpG sites (Table S4A in File S1) and genes whose expressions were significantly altered (Table S7 in File S1) (adjusted p value of <0.1 and log2 ratio>1) when comparing EOPET samples with controls. It should be noted, however, that gene expression and DNA methylation analyses were not performed on the same samples but, rather, phenotypically similar (EOPET) samples.

### Differentially Methylated Genes are not Strongly Correlated Between Independent Studies

When comparing CpG sites whose methylation patterns are altered in preeclampsia versus normal controls, we found very minimal overlap between our own results and those that have been previously published [16], [17]. This was the case regardless of how gender bias was controlled (either by treating male and female pregnancies separately or removing X chromosome data points). Specifically, in 2010, Robinson *et al.* [16] reported 34 differentially methylated CpG sites when comparing 4 normal with 4 EOPET tissue samples using the Humanmethylation27 Infinium microarray. In a larger study, the same group reported 282 differentially methylated CpG sites within 248 distinct genes when similar comparisons were made [17]. In our analysis of EOPET samples versus normal controls we could find only 1 (CHI3L2) and 2 (PAPPA2 and DAPK3) genes, respectively, that overlapped with these genes identified in these studies [16], [17]. Notably, direct comparison between these two previous data sets resulted in the identification of only 2-shared CpG sites in 2 distinct genes (MEST and TIMP3). Furthermore, we could find only 5 genes (DAPK3, DUSP1, PAPPA2, ITCH, DENND2D) that overlapped with our gender-specific analysis of our data of all early and late combined preeclampsia samples (Tables 2 and 3).

### Gender-Specific Differences in Placental DNA Methylation Provide Positive Control Data

**Figure 3:**
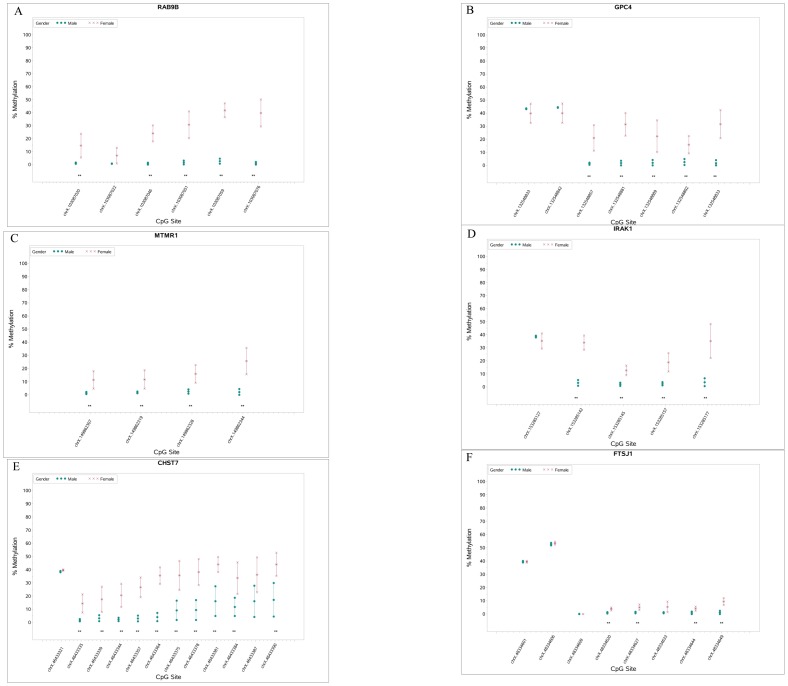
Targeted Analysis of Fetal Gender-Specific Differences in Placental DNA Methylation. Locus-specific analysis of CpG methylation was carried out by high-throughput bisufite sequencing. Statistically significant differences in% methylation between samples are marked with **. Genomic coordinates corresponding to values on the x-axis can be found in Table S6B in File S1.

Given the lack of correlation between independent studies and the relatively modest differences in methylation frequency at specific loci in our data, we compared male versus female pregnancies with the goal of identifying X chromosome-specific alterations in DNA methylation associated with dosage compensation and X inactivation. We anticipated that X chromosome-specific probes could effectively serve as positive controls to establish the sensitivity of the microarray platform. As predicted by the clustering data shown in Figure 1, we identified numerous differentially methylated sites on the X chromosome regardless of whether male versus female comparisons concerned normal or preeclampsia samples. A subset of these gender-specific methylation differences was confirmed by Sequenom Epityper analysis, and a further subset was confirmed by high-throughput targeted bisulfite sequencing (Figure 3). A number of gender-specific differentially methylated CpG sites were also found on autosomes (Table S8A and S8B in File S1). These differences were generally quite small but this finding supports the fact that male and female samples can be broadly separated by unsupervised hierarchical clustering and multidimensional scaling when analyzing both preeclampsia samples (Figures S2A and S2B) and normal controls (Figures S2C and S2D).

### Reanalysis of Previously Published Data Reveals Impact of Statistical and Bioinformatic Methods

The lack of overlap between genes identified in the current study and previously published reports may either be the result of biological variation, study design and/or statistical analysis of data. We therefore downloaded raw data from one of these manuscripts and re-analyzed it using our own statistical pipeline to test whether fundamental differences in analytical approach can dramatically influence results. Of 248 distinct genes identified previously [17] only 56 were found to overlap with CpG sites whose altered DNA methylation was statistically significant in our analysis of the same data (Table S5A in File S1). Additionally, we identified CpG sites within 75 distinct genes that were not identified in the original study (Table S5B in File S1). Notably, when we compared two previously published data sets that compared placental tissue from EOPET with normal controls [16], [17] we found only 3 genes whose DNA methylation patterns were identified as significant (TIMP3, MEST and CXCL9) out of a maximum possible 34.

## Discussion

In this study, we describe DNA methylation patterns in human placental tissue samples obtained at birth from both preeclamptic and normal pregnancies. In addition to identifying disease-specific alterations in placental methylation, we also identified gender-dependent changes that are both X-chromosome and autosome-specific. We could find no correlation between promoter DNA methylation and gene expression for differentially methylated CpG sites, although gene expression and DNA methylation analyses were not performed on the same samples but, rather, phenotypically similar (EOPET) samples.

Furthermore, we identified a significant lack of correlation between multiple independent data sets from three separate studies.

It is notable that we identified minimal overlap between our data and two previously published studies describing differences between normal controls and EOPET [16], [17]. One reason for this may be that, to control for the documented influence of tobacco smoke on DNA methylation, our study used only placental samples from self-reported non-smokers. This may be significant given the fact that smoking has been shown to influence DNA methylation in a variety of contexts [23], [24], [25], [26], [27], [28], [29]. Similarly, given that DNA methylation is also known to be affected by a variety of environmental stresses [30] the lack of consistency between independent studies may also be due to other complex variables. Furthermore, preeclampsia is an extremely complex syndrome that is classified by gross phenotypic parameters. Therefore it is possible that, despite exhibiting similar clinical characteristics, there are significant phenotypic differences between the patients in our cohort and those of other studies that may influence DNA methylation patterns. Furthermore, statistical methods are likely to influence results such that different statistically significant CpG sites are identified in the same data when different algorithms are used. Indeed the fact that our re-analysis of existing data resulted in the identification of many new genes is likely due to the fact that our data were analyzed using the Limma algorithm and previously published data were analyzed by SAM. Finally, our control samples, unlike these previous studies, were not selected from mothers who underwent premature labor to provide gestational age matching for early onset preeclampsia cases. We specifically chose not to use premature labor controls to avoid possible complications of using non-normal samples and confounding our results. Our approach is supported by our data, which show no clear association between DNA methylation patterns and gestational age within the gestational age range (third trimester) of samples used.

A number of notable differences in DNA methylation of specific genes were observed in our data. For example, the expression of Purinergic Receptor P2X, Ligand-Gated Ion Channel, 4 (P2RX4) has been shown to be elevated in both pre-term and term placental samples from preeclampsia cases compared to normal controls. CpG methylation within the P2RX4 promoter was reduced in preeclampsia samples relative to controls in our data, suggesting elevated transcription [31]. Similarly, a CpG site in the promoter region of Pregnancy-Associated Plasma Protein A2 (PAPPA2), whose expression is elevated in preeclampsia and is a promising biomarker for its detection [32], [33], [34], [35], exhibited reduced DNA methylation in preeclampsia samples compared to normal controls.

It is somewhat surprising that our gender-specific analysis of preeclampsia-related changes in DNA methylation (Tables 2 and 3) identified significant differences in only pregnancies involving female fetuses. Although there is no prior evidence that preeclampsia-specific DNA methylation patterns are gender-specific, there are numerous examples of gender-specific DNA methylation, chromatin structure and gene expression patterns involving autosomal loci. For example, it has been shown that environmentally-dependent modifications of histone H3 are sexually dimorphic in the developing mouse brain [36], and that patterns of acetylation (but not methylation) can be masculinized in females by testosterone in utero [37]. Furthermore, dimorphic gene expression has been reported in liver, kidney, blastocysts, lacrimal gland, placenta and brain [38], [39], [40], [41], [42] and sex-specific methylation patterns have been previously characterized [43], [44].

It is not surprising that the inclusion of X chromosome-specific data resulted in clear gender-dependent hierarchical clustering patterns. Notably, when X chromosome data were removed, samples were still broadly separated into gender-associated clusters and statistically significant gender-dependent autosomal alterations in DNA methylation were observed. These observations are worthy of further study at higher resolution with a larger sample size. The identification of autosomal origins of gender-specific differences is significant because previous studies of DNA methylation in preeclampsia have controlled for fetal gender by simply removing X chromosome specific probes from the analyses [16], [17]. Based on our observations, this approach may not be sufficient to control for gender bias in DNA methylation.

In summary, we have undertaken a systematic analysis of preeclampsia-associated DNA methylation changes in the placenta. We identified a number of differentially methylated CpG sites in tissues from preeclampsia patients including a number that were altered only in EOPET cases compared to controls.

## Supporting Information

Figure S1 **Hierarchical clustering, with respect to gestational age at delivery, of DNA patterns in placental tissues from preeclampsia patients.**
(TIF)

Figure S2 A. Hierarchical clustering, with respect to fetal gender, of DNA patterns in placental tissues from preeclampsia patients. B. MDS Analysis, with respect to fetal gender of DNA patterns, in placental tissues from preeclampsia patients. C. Hierarchical clustering with respect to fetal gender of DNA patterns in placental tissues from normal controls. D. MDS Analysis with respect to fetal gender of DNA patterns in placental tissues from normal controls.
(TIFF)

File S1 **Tables S1–S8.** Table S1A - Bisulfite Sequencing Primers and Tm's. Table S1B- PCR reaction conditions used. Table S1C. Epityper MassARRAY Primer Sequences. Table S2A-Preeclampsia-Associated Changes in Placental DNA Methylation Without Control for Fetal Gender: CpG sites at which samples from preeclampsia samples are more highly methylated than those from normal controls are shown. Table S2B- Preeclampsia-Associated Changes in Placental DNA Methylation Without Control for Fetal Gender: CpG sites at which samples from normal controls are more highly methylated than those from affected individuals are shown. Table S3A- Fetal Gender-Dependent Preeclampsia-Associated Changes in Placental DNA Methylation: CpG sites at which samples from normal controls are more highly methylated (<10%) than those from affected individuals are shown. Table S3B- Fetal Gender-Dependent Preeclampsia-Associated Changes in Placental DNA Methylation: CpG sites at which samples from preeclampsia samples are more highly methylated (<10%) than those from normal controls are shown. Table S4A- DNA Methylation Changes in Early Onset Preeclampsia (EOPET) Versus Normal Controls.% methylation levels for all significant differentially methylated CpG sites in EOPET (PE<34 weeks, n = 9) and normal controls (norm, n = 24). Adjusted p values are shown with FDR controlled at 10%. Table S4B- DNA Methylation Changes in Early Onset Preeclampsia (EOPET) Adjusted p values are shown with FDR controlled at 10%. Table S5A- Genes previously identified as containing differentially methylated CpG sites (reference 17) that were confirmed using our statistical/infomratic piepline. Table S5B- Genes identified from published data (reference 17) using our statistical/informatic pipeline containing differentially methylated CpG sites that were not previously identifed. Table S6A- Genomic Coordinates of Targeted DNA Methylation Analsysis Loci. Table S6B- Genomic Coordinates of Targeted DNA Methylation Analsysis Loci. Table S7- Gene expression differences between EOPET and normal placental tissue samples. Table S8A- Gender specific differences in DNA methylation at autosomal loci in normal samples.
(XLSX)
