# Supplementary figures and images for: Comprehensive Analysis of Preeclampsia-Associated DNA Methylation in the Placenta
Source: PLoS One. 2014 Sep 23;9(9):e107318. doi: 10.1371/journal.pone.0107318 (PMC4172433; doi:10.1371/journal.pone.0107318)

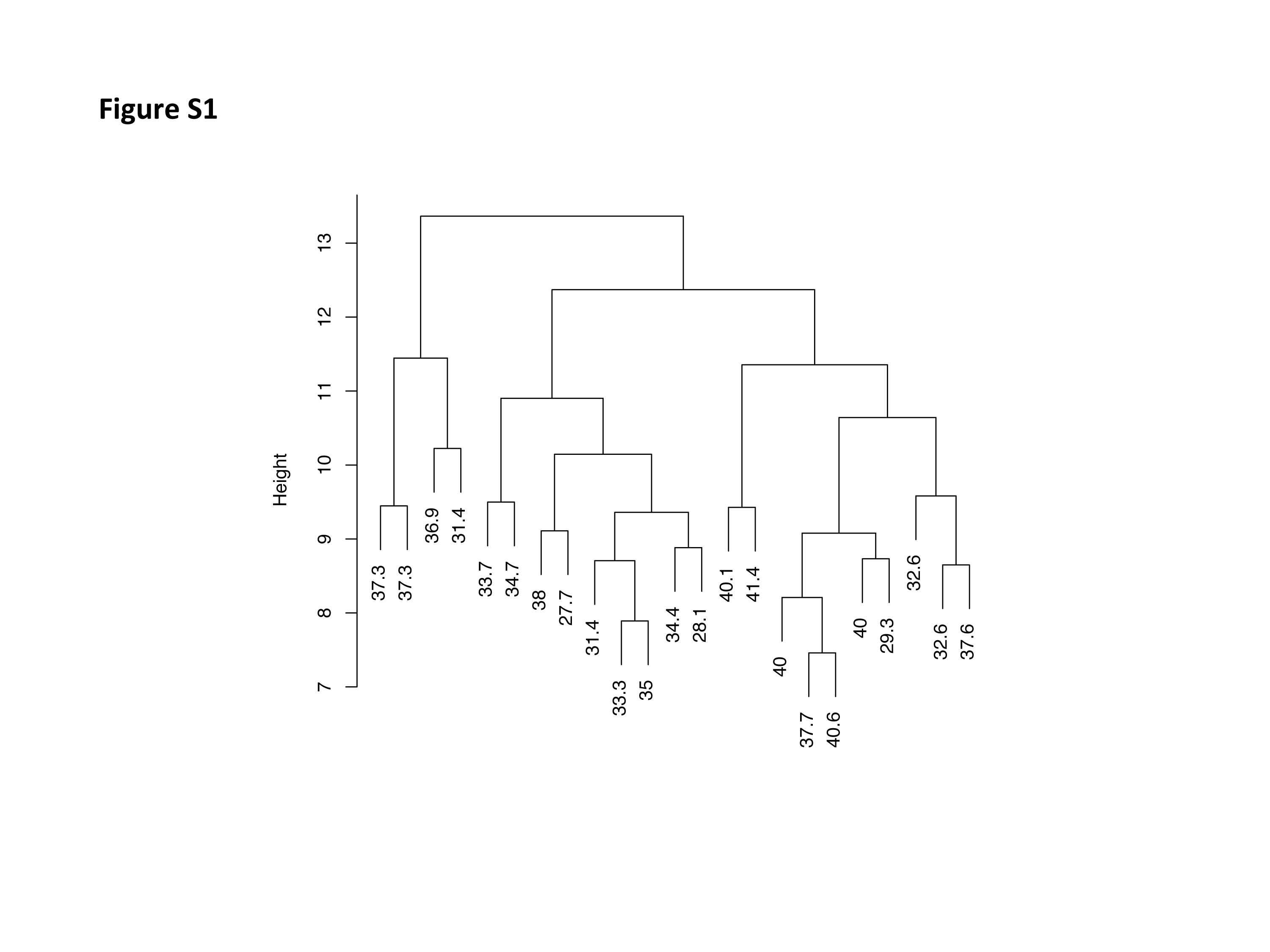

Supplement: Figure S1 — Hierarchical clustering, with respect to gestational age at delivery, of DNA patterns in placental tissues from preeclampsia patients. (TIF) [file pone.0107318.s001.tif]

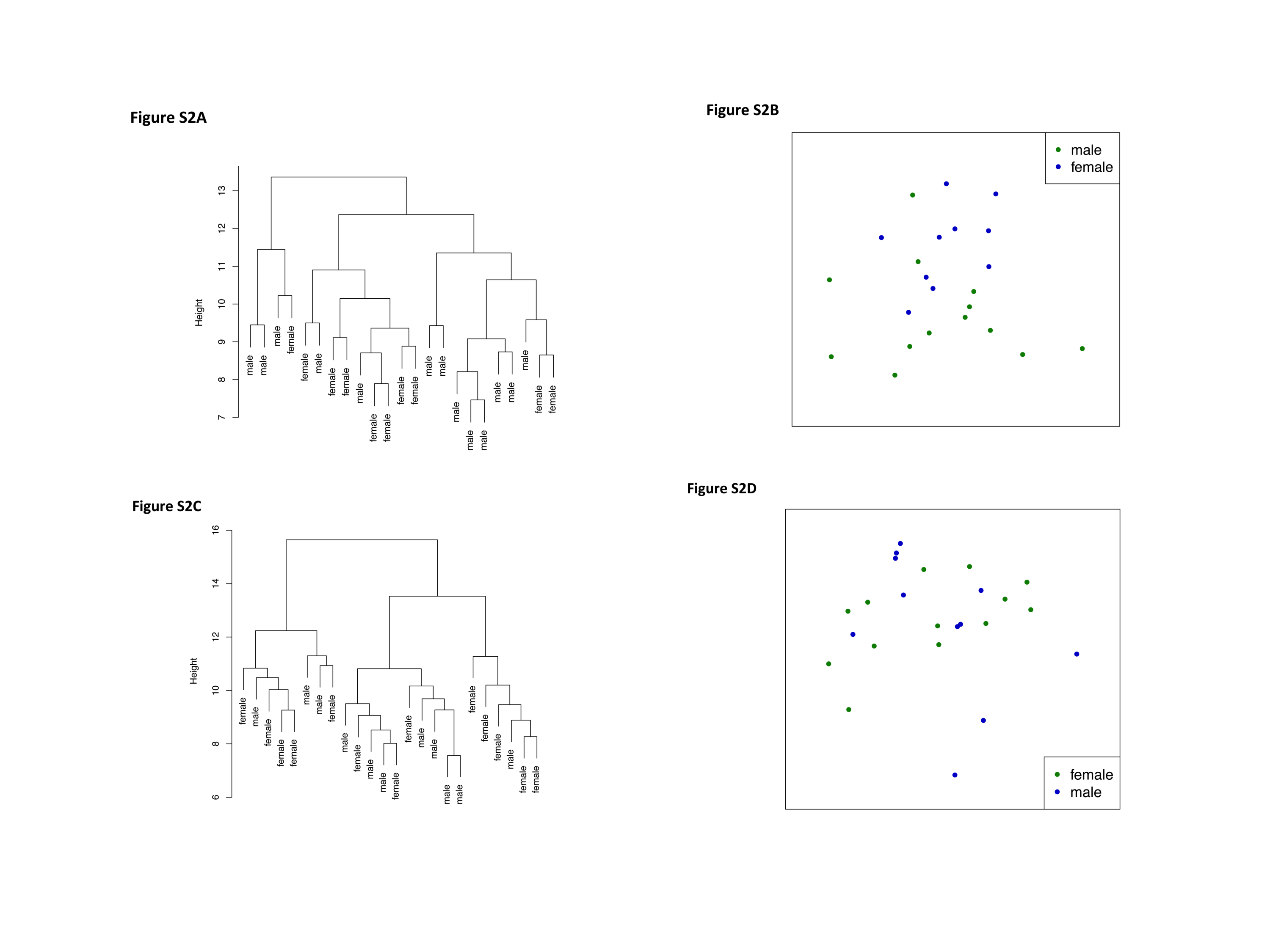

Supplement: Figure S2 — A. Hierarchical clustering, with respect to fetal gender, of DNA patterns in placental tissues from preeclampsia patients. B. MDS Analysis, with respect to fetal gender of DNA patterns, in placental tissues from preeclampsia patients. C. Hierarchical clustering with respect to fetal gender of DNA patterns in placental tissues from normal controls. D. MDS Analysis with respect to fetal gender of DNA patterns in placental tissues from normal controls. (TIFF) [file pone.0107318.s002.tiff]
